# Supplementary material for: Heterospin frustration in a metal-fullerene-bonded semiconductive antiferromagnet
Source: Nat Commun. 2022 Jan 25;13:495. doi: 10.1038/s41467-022-28134-w (PMC8789904; doi:10.1038/s41467-022-28134-w)
Supplement: Supplementary file 1 — Supplementary Information [file 41467_2022_28134_MOESM1_ESM.pdf]

Supplementary information

Heterospin Frustration in a Metal-Fullerene-Bonded Semiconductive Antiferromagnet

Yongbing Shen,[1,*] Mengxing Cui,[1] Shinya Takaishi,[1] Hideyuki Kawasoko,[1] Kunihisa Sugimoto,[2] Takao Tsumuraya,[3] Akihiro Otsuka,[4,5] Eunsang Kwon,[6] Takefumi Yoshida,[1] Norihisa Hoshino,[7] Kazuhiko Kawachi,[8] Yasuhiko Kasama,[8] Tomoyuki Akutagawa,[7] Tomoteru Fukumura,[1,9] and Masahiro Yamashita[1,10,*]

[1] Department of Chemistry, Graduate School of Science, Tohoku University, 6-3 Aza-Aoba, Aramaki, Sendai 980-8578, Japan

[2] Diffraction & Scattering Division Synchrotron Radiation Research Institute 679-5198 Hyogo, Japan

[3] Priority Organization for Innovation and Excellence, Kumamoto University, 2-39-1 Kurokami, Kumamoto 860-8555, Japan

[4] Division of Chemistry, Graduate School of Science, Kyoto University, Sakyo-Ku, Kyoto 606-8502, Japan

[5] Research Center for Low Temperature and Materials Sciences, Kyoto University, Sakyo-Ku, Kyoto 606-8501, Japan

[6] Research and Analytical Center for Giant Molecules, Tohoku University, 6-3, Aramaki-Aza-Aoba, Aoba-ku, Sendai 980-8578, Japan

[7] Institute of Multidisciplinary Research for Advanced Materials, Tohoku University,

2-1-1 Katahira, Aoba-Ku, Sendai 980-8577, Japan

[8] Idea International Co., Ltd., 1-15-35 Sagigamori, Aoba-ku, Sendai 981-0922, Japan

[9] Advanced Institute for Materials Research and Core Research Cluster, Tohoku University, Sendai 980-8577, Japan

[10] School of Materials Science and Engineering, Nankai University, Tianjin 300350, China

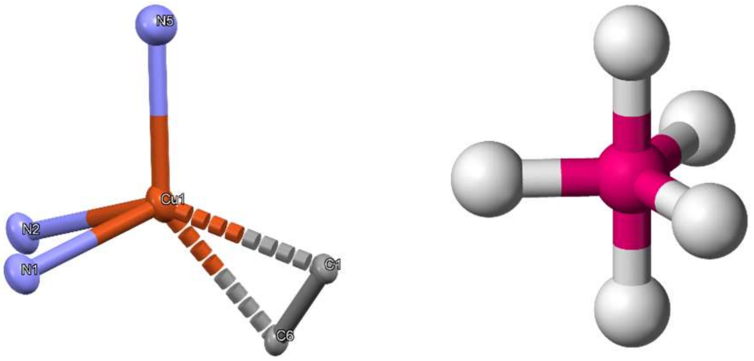

Supplementary Figure 1. The coordination geometry of $Cu(N)_3(C)_2$ (left) and PF_5, D_{3h} symmetry(right)

∠N5Cu1N2 = 104.1˚   ∠N5Cu1N1 = 104.1˚

∠N5Cu1C1 = 109.0˚   ∠N5Cu1C1 = 141.9˚

So, deviation parameters P are:

P1 = (104.1-90) / 90 *100% = 15.6%

P2 = (109-90) / 90 *100% = 21.1%

P3 = (180-140) / 180 *100% = 22.2%

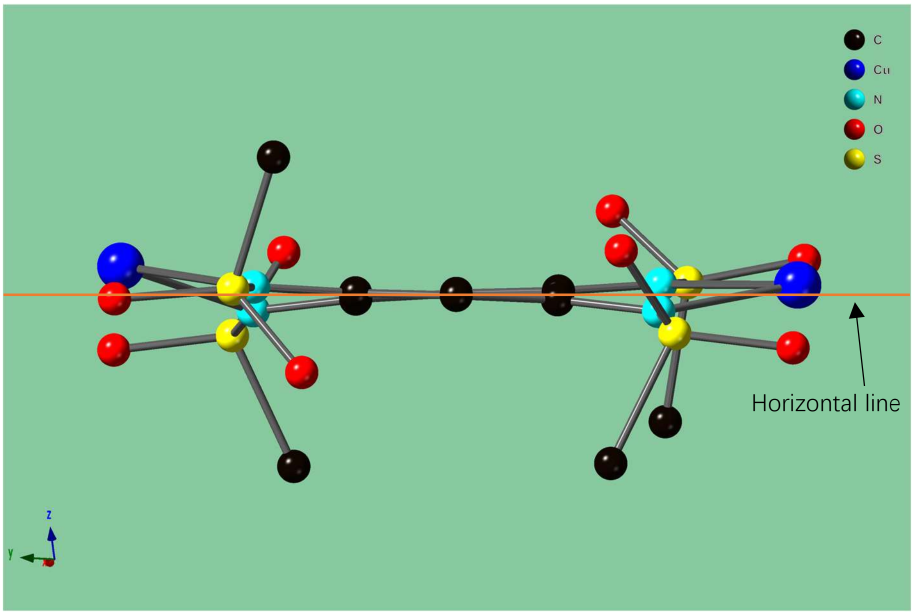

Supplementary Figure 2. The geometry and the shape of the dinuclear metal complex $Cu_2(L)$. The central benzene ring is slightly distorted.

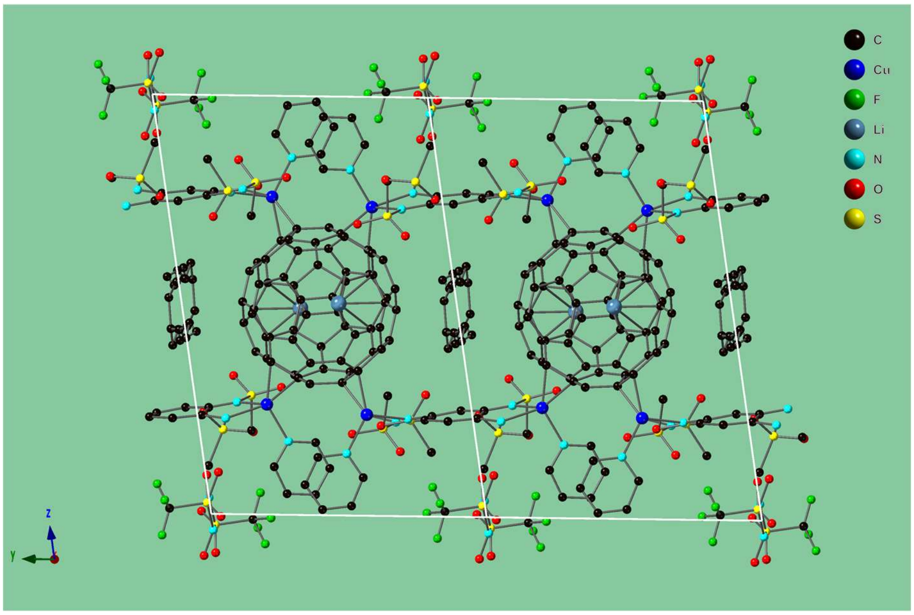

Supplementary Figure 3. The arrangement of the disordered hexane molecules and the counter anion NTf_2^-. The hexane molecules are trapped in the center of the square geometry of $[Cu_2(L)(py)_2Li^+@C_{60}]_2$. The NTf_2^- anions locate near the ligand.

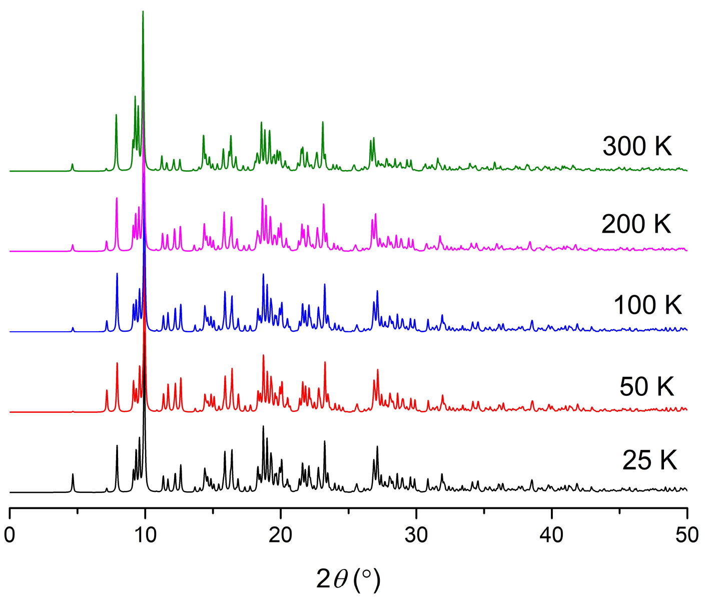

Supplementary Figure 4. The simulated PXRD patterns at 25, 50, 100, 200 and 300 K.

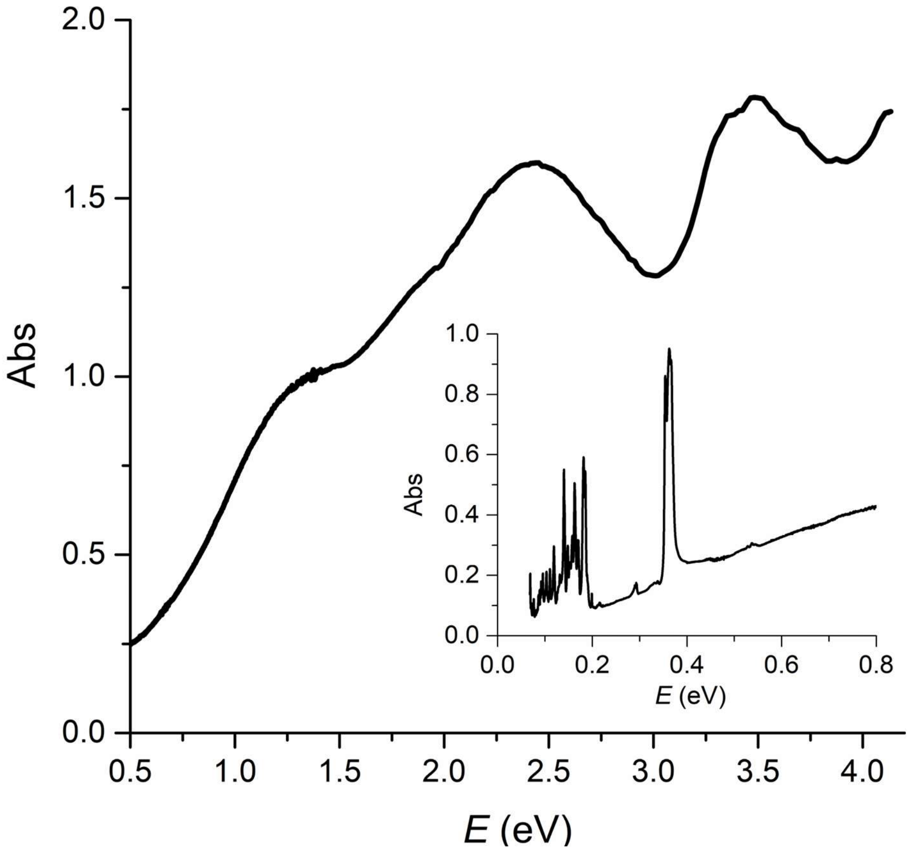

Supplementary Figure 5. The solid-state absorption spectrum of **1** at room temperature by using the KBr pellet method, insert is the absorbance in the IR region.

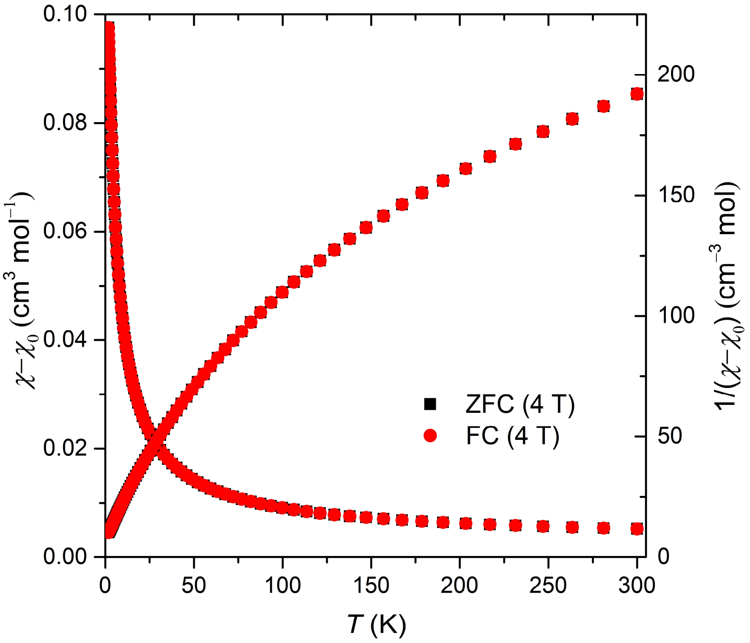

Supplementary Figure 6. The temperature-dependence of magnetic susceptibility (χ-χ_0) in 1.8–300 K under FC and ZFC in 4 T filed. The black curve represents the best fit by considering the possible exchange interactions between Cu^{2+} ions and $Li^+@C_{60}^{\bullet-}$. Inset: The $(\chi$-$\chi_0)^{-1}$–T plot.

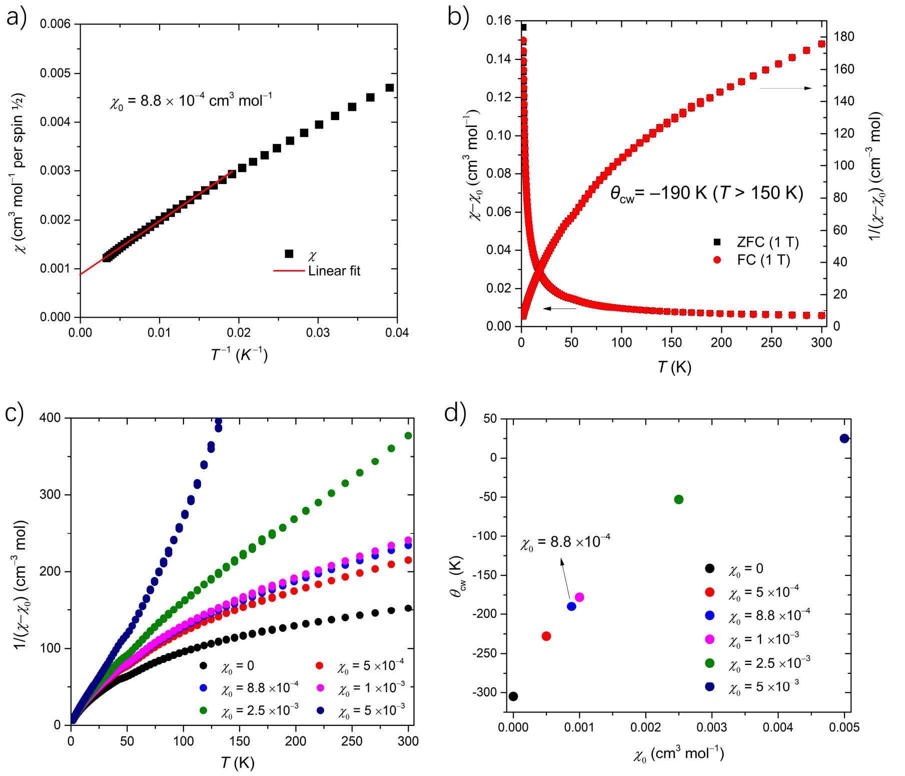

Supplementary Figure 7. a) The χ versus T^{-1} plot, χ_0 was determined by linear extrapolation of the χ versus T^{-1} plot down to zero K. b) The $(\chi-\chi_0)$ versus T plot and its $(\chi-\chi_0)^{-1}$ versus T plot. c) $(\chi-\chi_0)^{-1}$ versus T plot at various χ_0. d) temperature dependence of Curie-Weiss temperature at various χ_0.

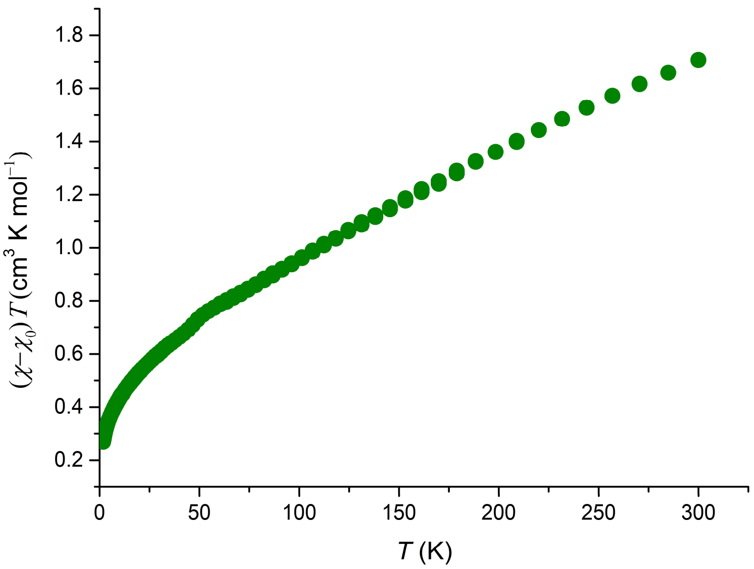

Supplementary Figure 8. The $(\chi-\chi_0)T$–T plot of **1** in 1 T field in 1.8-300 K. The fast decrease of $(\chi-\chi_0)T$ as a reduction of temperature indicates the presence of antiferromagnetic interaction in **1**.

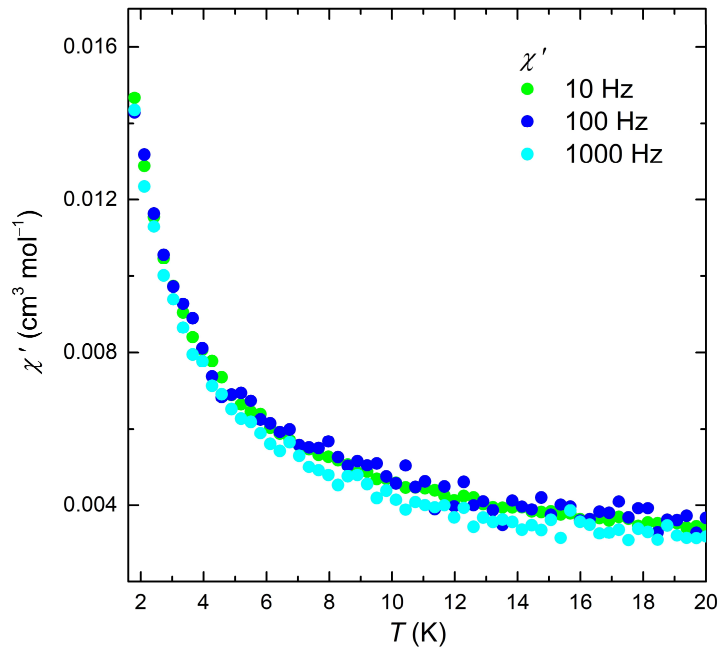

Supplementary Figure 9. Temperature dependence of in-phase magnetic susceptibility (χ') at various frequencies.

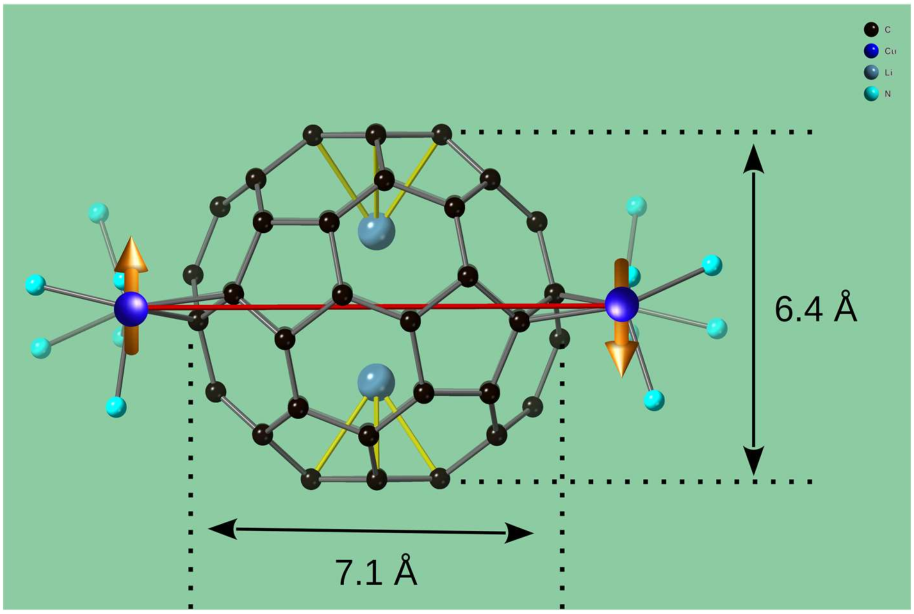

Supplementary Figure 10. The $Li^{+}@C_{60}$ cage in **1** is geometrically distorted with diagonal lengths of 6.4 and 7.1Å. The adjacent two Cu^{2+} ions interact antiferromagnetically.

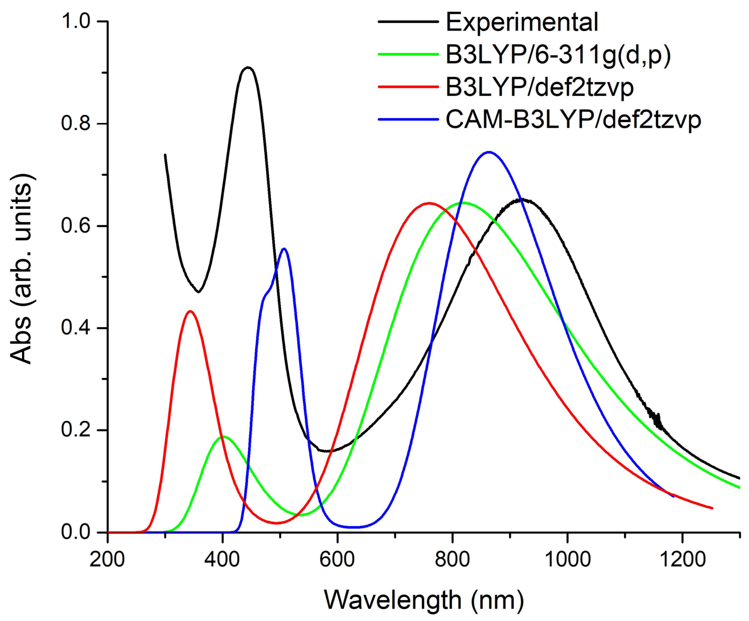

Supplementary Figure 11. The experimental (black) and simulated (the other colors) absorbance spectra of $Cu_2(L)(py)_4$ in dichloromethane solution in the energy length of 300-1500 nm. Two strong absorbance bands are observed at 920 and 443 nm for the experimental.

we tried the TD-DFT calculation by using CAM-B3LYP/de2tzvp for $Cu_2(L)(py)_4$. The electron transitions from HOMO to LUMO were observed at 920, 765, 760, 860 nm by using experimental, B3LYP/6-311G(d,p), B3LYP/def2tzvp and CAM-B3LYP/def2tvp, respectively. The results are summarized in the table and figures. From the results, CAM-B3LYP/de2tzvp method showed better results.

Supplementary Table 1. Absorbance bands in various calculation methods.

**Basis sets**	**band (1)**	**band (2)**
Experimental	920 nm	443 nm
B3LYP/6-311G(d,p)	765 nm	412
B3LYP/def2tzvp	760 nm	340 nm
CAM-B3LYP/de2tzvp	860 nm	470 nm, 507 nm

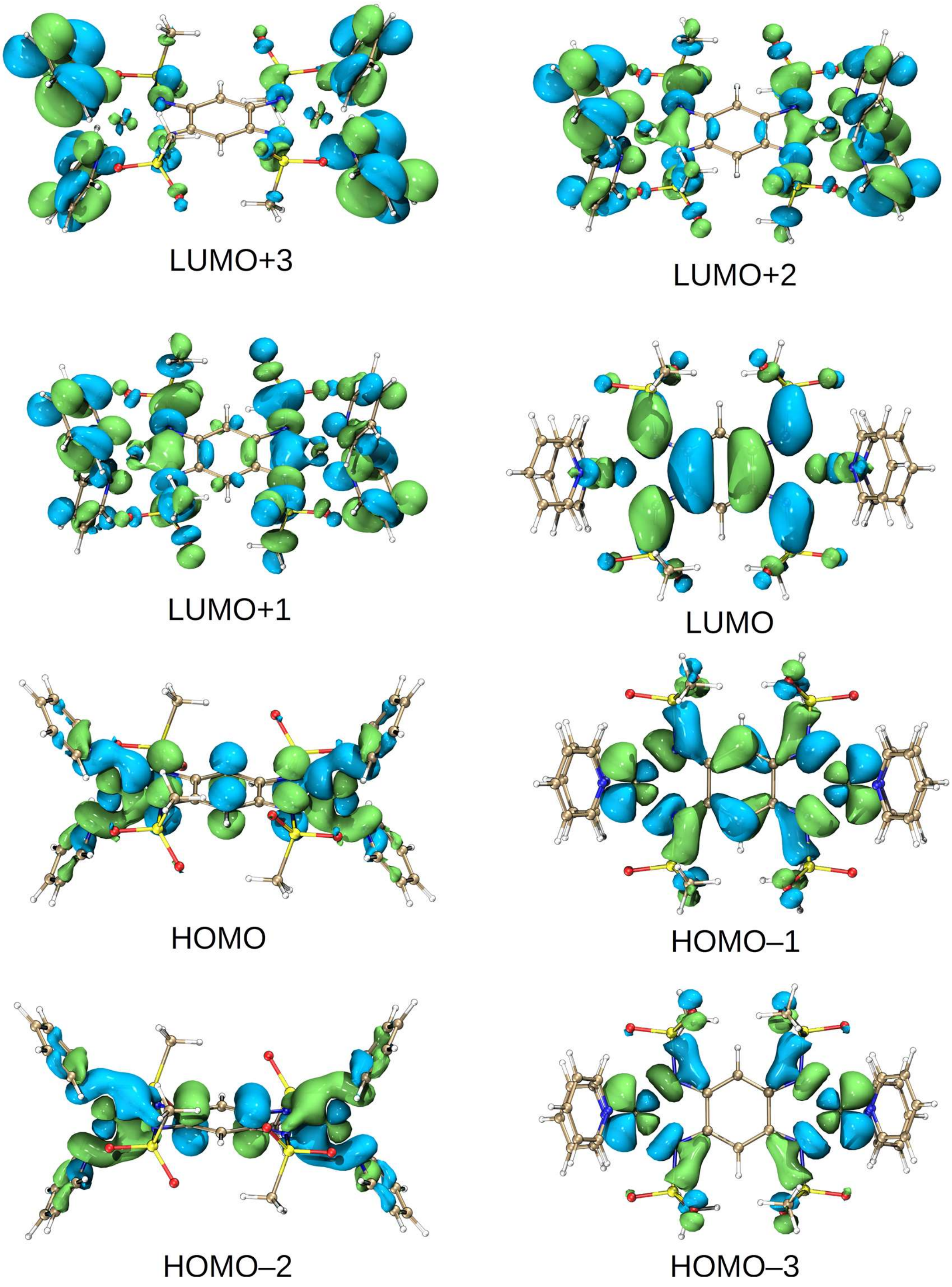

Supplementary Figure 12. The selected frontier molecular orbitals of $Cu_2(L)(py)_4$. The absorbance band of $Cu_2(L)(py)_4$ at 443 nm with the gap of 2.80 eV corresponds to the ET from HOMO–1 to LUMO+1.

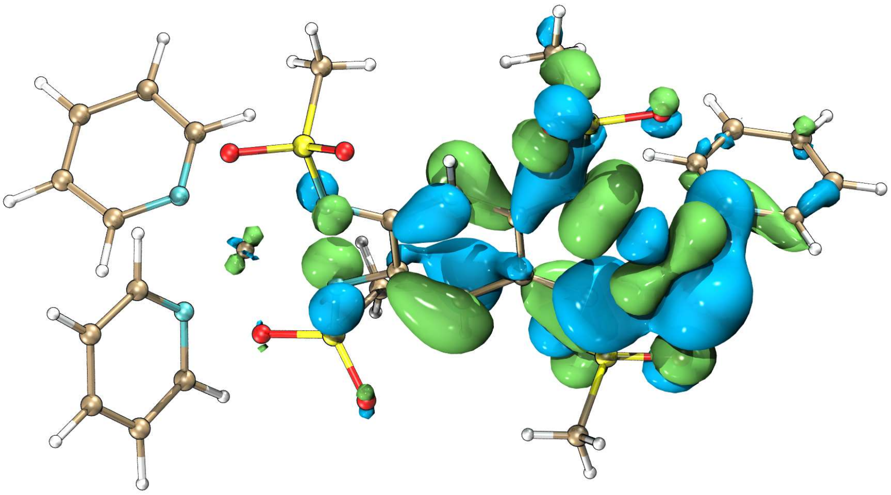

Supplementary Figure 13. The electron density on the HOMO–1 of the open-shell of $Cu_2L(py)_3$, which contributed 4% electrons to the HOMO of the complex. The electrons mainly delocalised on the right side of the ligand and Cu^{2+} ion.

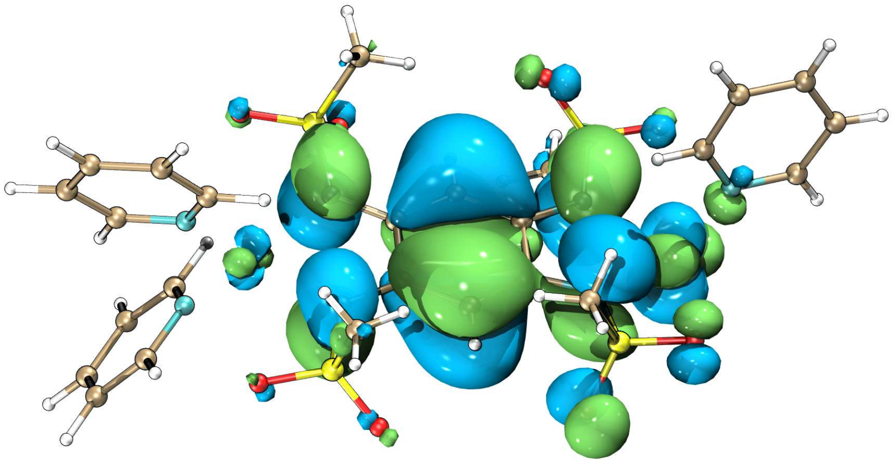

Supplementary Figure 14. The electron density on the HOMO–2 of the open-shell of $Cu_2L(py)_3$, which contributed 2% electrons to the HOMO of the complex. The electrons mainly located on the central ligand and the right side of the Cu^{2+} ion.

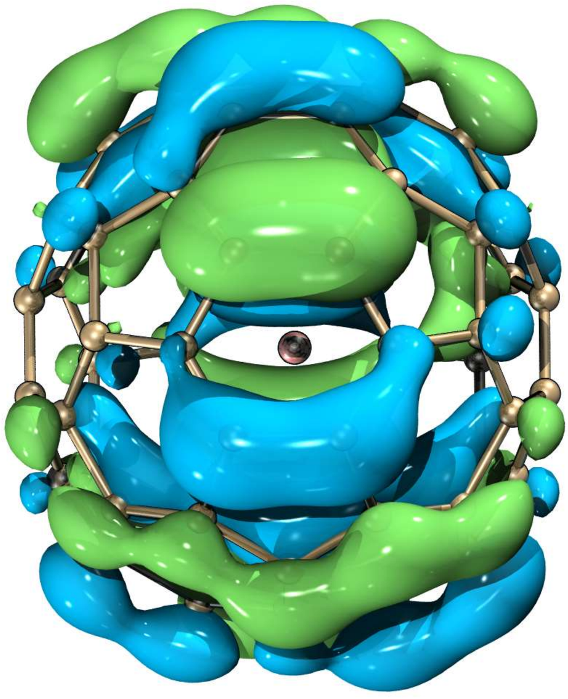

Supplementary Figure 15. The electron density on the HOMO–1 of the open-shell of $Li^{+}@C_{60}^{\bullet-}$.

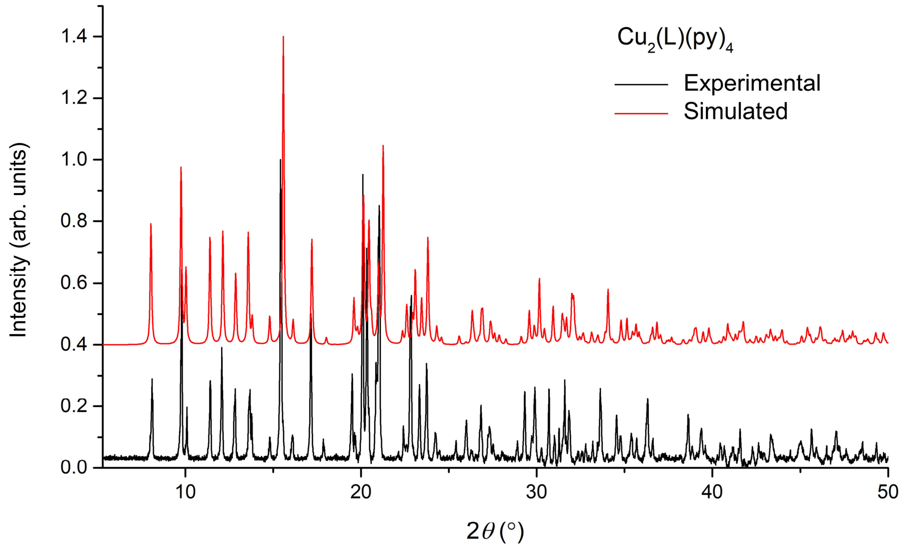

Supplementary Figure 16. PXRD patterns for $Cu_2(L)(py)_4$.

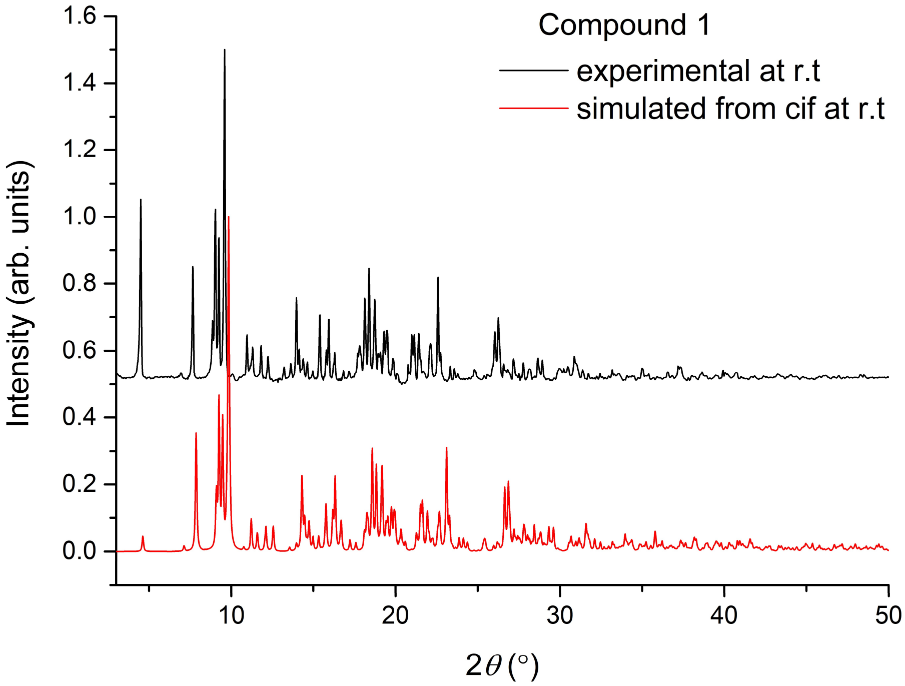

Supplementary Figure 17. PXRD patterns for **1**.

Supplementary Table 2. Generalized charge decomposition analysis (GCDA) for triplet $Cu_2(L)(py)_3Li^+@C_{60}$.

============= Charge decomposition analysis (CDA) result =============

d = The number of electrons donated from fragment 1 to fragment 2

b = The number of electrons back donated from fragment 2 to fragment 1

r = The number of electrons involved in repulsive polarization

**** Result for alpha electrons ****

**Orb.**	**Occ.**	**d**	**b**	**d - b**	**r**
1	1.000000	-0.000000	-0.000000	-0.000000	-0.000000
2	1.000000	-0.000015	0.000028	-0.000043	0.000011
3	1.000000	-0.000000	-0.000000	-0.000000	-0.000000
4	1.000000	-0.000000	-0.000000	-0.000000	-0.000000
5	1.000000	-0.000001	0.000000	-0.000001	0.000000
6	1.000000	-0.000000	0.000002	-0.000002	-0.000000
7	1.000000	-0.000000	-0.000000	-0.000000	-0.000000
8	1.000000	-0.000599	-0.001569	0.000970	-0.000865
9	1.000000	-0.000000	-0.000000	-0.000000	-0.000000
10	1.000000	-0.000000	-0.000000	-0.000000	-0.000000
11	1.000000	-0.000000	-0.000000	-0.000000	-0.000000
12	1.000000	-0.000214	-0.000135	-0.000079	-0.000150
13	1.000000	-0.000057	-0.000095	0.000039	-0.000034
14	1.000000	-0.000018	-0.000034	0.000015	-0.000006
15	1.000000	-0.000002	-0.000001	-0.000001	-0.000000
16	1.000000	-0.000000	-0.000000	-0.000000	-0.000000
17	1.000000	-0.000001	-0.000000	-0.000001	-0.000000
18	1.000000	-0.000002	-0.000001	-0.000001	-0.000000
19	1.000000	-0.000008	-0.000002	-0.000006	-0.000001
20	1.000000	-0.000022	-0.000047	0.000025	-0.000003
21	1.000000	-0.000025	0.000002	-0.000027	-0.000002
22	1.000000	-0.000021	-0.000073	0.000051	0.000002
23	1.000000	-0.000000	-0.000000	-0.000000	-0.000000
24	1.000000	-0.000000	-0.000000	-0.000000	-0.000000
25	1.000000	-0.000076	-0.000181	0.000106	-0.000025
26	1.000000	-0.000001	-0.000000	-0.000001	-0.000000
27	1.000000	-0.000001	-0.000000	-0.000000	-0.000000
28	1.000000	-0.000041	-0.000168	0.000127	-0.000018
29	1.000000	-0.000056	-0.000028	-0.000028	-0.000006
30	1.000000	-0.000000	-0.000000	-0.000000	-0.000000
31	1.000000	-0.000000	-0.000000	-0.000000	-0.000000
32	1.000000	-0.000000	-0.000000	-0.000000	-0.000000
33	1.000000	-0.000000	-0.000000	-0.000000	-0.000000

34	1.000000	-0.000005	-0.000001	-0.000004	-0.000001
35	1.000000	-0.000017	-0.000000	-0.000017	0.000000
36	1.000000	-0.000003	0.000000	-0.000003	-0.000000
37	1.000000	-0.000000	-0.000000	-0.000000	-0.000000
38	1.000000	-0.000047	0.000010	-0.000057	-0.000002
39	1.000000	-0.000000	-0.000000	-0.000000	-0.000000
40	1.000000	-0.000000	-0.000000	-0.000000	-0.000000
41	1.000000	-0.000000	-0.000000	-0.000000	-0.000000
42	1.000000	-0.000000	-0.000000	-0.000000	-0.000000
43	1.000000	-0.000000	-0.000000	-0.000000	-0.000000
44	1.000000	-0.000017	-0.000006	-0.000011	-0.000003
45	1.000000	-0.000007	-0.000001	-0.000006	-0.000001
46	1.000000	-0.000001	-0.000000	-0.000000	-0.000000
47	1.000000	-0.000001	-0.000001	-0.000000	-0.000000
48	1.000000	-0.000001	-0.000000	-0.000000	-0.000000
49	1.000000	-0.000003	-0.000001	-0.000003	-0.000001
50	1.000000	0.000745	-0.000031	0.000775	-0.000012
51	1.000000	-0.001659	-0.000086	-0.001574	-0.000208
52	1.000000	-0.000018	-0.000002	-0.000017	-0.000003
53	1.000000	-0.000003	-0.000001	-0.000001	-0.000001
54	1.000000	-0.000001	-0.000000	-0.000000	-0.000000
55	1.000000	-0.000001	-0.000000	-0.000001	-0.000000
56	1.000000	-0.000204	-0.000013	-0.000191	-0.000022
57	1.000000	-0.000245	-0.000018	-0.000227	-0.000015
58	1.000000	-0.000000	-0.000000	0.000000	-0.000000
59	1.000000	-0.000004	-0.000002	-0.000001	-0.000002
60	1.000000	-0.000624	-0.000027	-0.000597	-0.000034
61	1.000000	-0.000010	-0.000002	-0.000007	-0.000002
62	1.000000	-0.000003	-0.000001	-0.000001	-0.000001
63	1.000000	-0.000001	-0.000001	-0.000000	-0.000001
64	1.000000	-0.000001	-0.000000	-0.000000	-0.000000
65	1.000000	-0.000000	-0.000000	-0.000000	-0.000000
66	1.000000	-0.000000	-0.000000	-0.000000	-0.000000
67	1.000000	-0.000178	-0.000018	-0.000160	-0.000012
68	1.000000	-0.000000	-0.000000	-0.000000	-0.000000
69	1.000000	-0.000003	-0.000000	-0.000003	-0.000000
70	1.000000	-0.000098	-0.000003	-0.000095	-0.000005
71	1.000000	-0.000002	-0.000001	-0.000001	-0.000000
72	1.000000	-0.000141	-0.000010	-0.000131	-0.000009
73	1.000000	-0.000000	-0.000000	-0.000000	-0.000000
74	1.000000	-0.000001	-0.000000	-0.000000	-0.000000
75	1.000000	-0.000002	-0.000001	-0.000000	-0.000001
76	1.000000	-0.000002	-0.000001	-0.000001	-0.000000
77	1.000000	-0.000275	-0.000028	-0.000248	-0.000024

78	1.000000	-0.000001	-0.000001	-0.000001	-0.000000
79	1.000000	-0.000000	-0.000000	-0.000000	-0.000000
80	1.000000	-0.000925	-0.000050	-0.000875	-0.000063
81	1.000000	-0.000002	-0.000001	-0.000001	-0.000001
82	1.000000	-0.000003	-0.000002	-0.000002	-0.000001
83	1.000000	-0.000001	-0.000000	-0.000001	-0.000000
84	1.000000	-0.000004	-0.000001	-0.000002	-0.000001
85	1.000000	-0.000001	-0.000001	-0.000000	-0.000001
86	1.000000	-0.000386	-0.000026	-0.000360	-0.000044
87	1.000000	-0.000164	-0.000005	-0.000159	-0.000006
88	1.000000	-0.000004	-0.000001	-0.000002	-0.000001
89	1.000000	-0.000016	-0.000005	-0.000011	-0.000003
90	1.000000	-0.000000	-0.000000	0.000000	-0.000000
91	1.000000	-0.000001	-0.000000	-0.000000	-0.000000
92	1.000000	-0.000000	-0.000000	-0.000000	-0.000000
93	1.000000	-0.000001	-0.000001	-0.000000	-0.000000
94	1.000000	-0.000003	-0.000001	-0.000001	-0.000001
95	1.000000	-0.000001	-0.000000	-0.000001	-0.000000
96	1.000000	-0.000003	-0.000001	-0.000002	-0.000001
97	1.000000	-0.000000	-0.000000	-0.000000	-0.000000
98	1.000000	-0.000002	-0.000001	-0.000002	-0.000000
99	1.000000	-0.000096	-0.000006	-0.000090	-0.000008
100	1.000000	-0.000000	-0.000000	0.000000	-0.000000
101	1.000000	-0.000000	-0.000000	0.000000	-0.000000
102	1.000000	-0.000002	-0.000002	-0.000001	-0.000001
103	1.000000	-0.000000	-0.000000	0.000000	-0.000000
104	1.000000	-0.000154	-0.000043	-0.000111	-0.000027
105	1.000000	-0.000013	0.000034	-0.000047	0.000002
106	1.000000	-0.000001	-0.000000	-0.000001	-0.000000
107	1.000000	-0.000005	-0.000002	-0.000003	-0.000001
108	1.000000	-0.000066	-0.000008	-0.000058	-0.000008
109	1.000000	-0.000012	-0.000002	-0.000010	-0.000002
110	1.000000	-0.000030	-0.000004	-0.000026	-0.000004
111	1.000000	-0.000002	-0.000001	-0.000001	-0.000000
112	1.000000	-0.000025	-0.000006	-0.000019	-0.000004
113	1.000000	-0.000000	-0.000000	-0.000000	-0.000000
114	1.000000	-0.000022	0.000020	-0.000042	0.000001
115	1.000000	-0.000000	-0.000000	-0.000000	-0.000000
116	1.000000	-0.000000	-0.000000	-0.000000	-0.000000
117	1.000000	-0.000007	0.000007	-0.000014	0.000002
118	1.000000	-0.000008	0.000066	-0.000074	-0.000007
119	1.000000	-0.000000	-0.000000	-0.000000	-0.000000
120	1.000000	-0.000000	-0.000000	-0.000000	-0.000000
121	1.000000	-0.000000	-0.000000	-0.000000	-0.000000

122	1.000000	-0.000001	-0.000000	-0.000000	-0.000000
123	1.000000	-0.000000	-0.000000	-0.000000	-0.000000
124	1.000000	-0.000000	-0.000000	-0.000000	-0.000000
125	1.000000	-0.000009	-0.000008	-0.000000	-0.000001
126	1.000000	-0.000076	-0.000024	-0.000052	-0.000012
127	1.000000	-0.000028	-0.000016	-0.000012	-0.000006
128	1.000000	-0.000012	-0.000003	-0.000009	-0.000002
129	1.000000	-0.000040	-0.000098	0.000057	-0.000018
130	1.000000	-0.000150	-0.000068	-0.000082	-0.000027
131	1.000000	-0.000000	-0.000000	-0.000000	-0.000000
132	1.000000	-0.000222	-0.003718	0.003496	-0.002201
133	1.000000	-0.000000	-0.000000	-0.000000	-0.000000
134	1.000000	-0.000000	-0.000000	-0.000000	-0.000000
135	1.000000	-0.000000	-0.000000	-0.000000	-0.000000
136	1.000000	-0.000006	-0.000141	0.000135	-0.000067
137	1.000000	-0.000002	-0.000026	0.000024	-0.000021
138	1.000000	-0.000002	-0.000069	0.000067	-0.000020
139	1.000000	-0.000005	-0.000000	-0.000005	-0.000000
140	1.000000	-0.000001	-0.000000	-0.000001	-0.000000
141	1.000000	-0.000003	0.000000	-0.000004	-0.000000
142	1.000000	-0.000103	-0.000390	0.000287	-0.000192
143	1.000000	-0.000061	0.000177	-0.000238	0.000066
144	1.000000	-0.000001	-0.000000	-0.000000	-0.000000
145	1.000000	-0.000000	-0.000000	-0.000000	-0.000000
146	1.000000	-0.000003	0.000001	-0.000004	0.000000
147	1.000000	-0.000001	-0.000001	-0.000001	-0.000000
148	1.000000	-0.000134	0.000248	-0.000382	-0.000011
149	1.000000	-0.000481	0.001625	-0.002106	0.000358
150	1.000000	-0.000004	-0.000795	0.000790	0.000202
151	1.000000	0.001189	0.000060	0.001128	0.000381
152	1.000000	-0.000027	-0.000724	0.000697	-0.000150
153	1.000000	0.003739	-0.000327	0.004066	0.001147
154	1.000000	0.001614	-0.000090	0.001704	0.000442
155	1.000000	0.003339	-0.000283	0.003621	0.001019
156	1.000000	-0.000960	-0.002386	0.001426	-0.001002
157	1.000000	-0.000002	-0.000090	0.000089	-0.000006
158	1.000000	0.000537	-0.000012	0.000549	0.000125
159	1.000000	0.003783	-0.000096	0.003879	0.000921
160	1.000000	0.000670	-0.000016	0.000686	0.000157
161	1.000000	0.007275	-0.000223	0.007498	0.001974
162	1.000000	0.002595	-0.000032	0.002627	0.000413
163	1.000000	-0.000243	-0.000692	0.000449	-0.000182
164	1.000000	0.001008	-0.000011	0.001019	0.000372
165	1.000000	0.001380	-0.000037	0.001417	0.000412

166	1.000000	0.003767	-0.000031	0.003797	0.000723
167	1.000000	-0.000000	-0.000000	-0.000000	0.000000
168	1.000000	-0.000000	-0.000000	-0.000000	-0.000000
169	1.000000	0.002968	-0.000111	0.003079	0.000909
170	1.000000	0.001407	-0.000028	0.001435	0.000357
171	1.000000	0.000944	-0.000037	0.000980	0.000252
172	1.000000	0.007866	-0.000707	0.008573	0.002778
173	1.000000	-0.000124	-0.000176	0.000052	-0.000458
174	1.000000	-0.000098	-0.000288	0.000190	-0.000138
175	1.000000	-0.000001	-0.000000	-0.000000	-0.000000
176	1.000000	-0.000001	-0.000003	0.000002	0.000000
177	1.000000	-0.000007	-0.000040	0.000033	0.000006
178	1.000000	0.001225	-0.000230	0.001455	0.001863
179	1.000000	-0.000473	-0.000154	-0.000318	0.000486
180	1.000000	0.001065	-0.000211	0.001276	-0.000122
181	1.000000	0.000387	-0.000030	0.000417	-0.000201
182	1.000000	0.005015	-0.000199	0.005214	0.001541
183	1.000000	0.000748	0.000021	0.000727	0.000372
184	1.000000	0.000664	-0.000022	0.000686	0.000917
185	1.000000	-0.000289	-0.000168	-0.000121	-0.000700
186	1.000000	-0.000179	-0.000396	0.000217	-0.000617
187	1.000000	0.009664	-0.000330	0.009993	0.003212
188	1.000000	0.000769	-0.000021	0.000789	0.000117
189	1.000000	0.000354	-0.000003	0.000357	0.000235
190	1.000000	0.001714	-0.000025	0.001739	0.000399
191	1.000000	-0.000014	-0.000179	0.000165	-0.000002
192	1.000000	-0.000114	-0.000258	0.000144	0.000005
193	1.000000	0.004470	-0.000144	0.004614	0.001692
194	1.000000	0.002693	-0.000085	0.002778	0.001077
195	1.000000	0.001840	-0.000097	0.001936	0.000832
196	1.000000	0.000783	-0.000013	0.000796	0.000459
197	1.000000	-0.000019	-0.000002	-0.000017	0.000057
198	1.000000	0.000979	-0.000067	0.001046	0.000975
199	1.000000	0.001252	-0.000160	0.001412	0.000620
200	1.000000	0.001228	-0.000135	0.001363	0.000555
201	1.000000	-0.000001	0.000001	-0.000001	0.000000
202	1.000000	-0.000020	-0.000333	0.000313	0.000011
203	1.000000	-0.000001	-0.000010	0.000009	-0.000000
204	1.000000	-0.000000	-0.000000	-0.000000	-0.000000
205	1.000000	-0.000000	-0.000000	-0.000000	-0.000000
206	1.000000	-0.000071	-0.000479	0.000408	0.000141
207	1.000000	-0.000245	-0.000166	-0.000079	0.001149
208	1.000000	0.000116	-0.000354	0.000470	0.000032
209	1.000000	0.003676	-0.000117	0.003793	0.001243

210	1.000000	0.001688	-0.000011	0.001698	0.000718
211	1.000000	-0.000045	-0.000158	0.000113	-0.000001
212	1.000000	0.004964	-0.000349	0.005313	0.002962
213	1.000000	0.000029	0.000009	0.000020	0.000395
214	1.000000	0.001094	-0.000163	0.001257	0.000912
215	1.000000	0.000418	0.000014	0.000403	0.000176
216	1.000000	0.000193	-0.000012	0.000205	0.000204
217	1.000000	0.000177	-0.000005	0.000182	0.000312
218	1.000000	0.001101	-0.000029	0.001131	0.001297
219	1.000000	0.000479	-0.000014	0.000492	0.000725
220	1.000000	0.000058	-0.000001	0.000059	0.000297
221	1.000000	-0.000057	-0.000090	0.000033	-0.000011
222	1.000000	0.001377	-0.000595	0.001973	0.000851
223	1.000000	-0.000374	-0.000648	0.000274	-0.000119
224	1.000000	-0.000418	-0.001095	0.000676	0.000064
225	1.000000	-0.000039	-0.000309	0.000271	0.001185
226	1.000000	-0.000015	-0.000031	0.000016	0.000007
227	1.000000	-0.000102	-0.000345	0.000244	0.000267
228	1.000000	0.000170	-0.000014	0.000184	0.000306
229	1.000000	0.001217	-0.000052	0.001269	0.001524
230	1.000000	0.001085	-0.000102	0.001187	0.002512
231	1.000000	0.000287	-0.000013	0.000300	0.000515
232	1.000000	-0.000208	-0.000626	0.000418	0.000253
233	1.000000	-0.000052	0.000016	-0.000069	0.000195
234	1.000000	-0.000047	-0.000224	0.000177	0.000743
235	1.000000	-0.000714	-0.000026	-0.000688	0.000862
236	1.000000	0.001335	-0.000017	0.001352	0.002569
237	1.000000	0.000000	0.000001	-0.000001	0.000002
238	1.000000	0.002273	-0.000220	0.002493	0.002297
239	1.000000	0.000455	-0.000048	0.000503	0.000435
240	1.000000	0.000099	0.000003	0.000096	0.000214
241	1.000000	0.000081	0.000012	0.000069	0.000608
242	1.000000	-0.000001	-0.000001	-0.000000	0.000002
243	1.000000	-0.000033	0.000256	-0.000289	0.001152
244	1.000000	0.000060	-0.000047	0.000107	0.000047
245	1.000000	0.001398	-0.000306	0.001704	0.001795
246	1.000000	0.000764	-0.000107	0.000870	0.001597
247	1.000000	-0.000163	-0.000473	0.000309	-0.000156
248	1.000000	-0.000026	-0.000049	0.000023	0.000019
249	1.000000	-0.000142	-0.000244	0.000102	0.000729
250	1.000000	0.000203	-0.000809	0.001012	0.003203
251	1.000000	0.000055	-0.000155	0.000210	0.000652
252	1.000000	0.001078	-0.000642	0.001720	0.002970
253	1.000000	0.000743	-0.000639	0.001382	0.002022

254	1.000000	0.002929	0.000029	0.002900	0.003126
255	1.000000	0.000139	0.000009	0.000130	0.000448
256	1.000000	-0.000005	-0.000013	0.000008	0.000036
257	1.000000	0.001562	-0.000355	0.001917	0.002295
258	1.000000	0.000803	-0.000022	0.000825	0.001957
259	1.000000	-0.000093	0.000114	-0.000207	0.000188
260	1.000000	-0.000409	-0.000681	0.000271	0.001682
261	1.000000	-0.000044	-0.000054	0.000009	0.000379
262	1.000000	0.000014	0.000021	-0.000007	0.001867
263	1.000000	0.000149	-0.000748	0.000897	0.007964
264	1.000000	-0.000033	0.000036	-0.000069	0.000188
265	1.000000	-0.000123	0.000066	-0.000189	0.000391
266	1.000000	0.000529	-0.000004	0.000534	0.000601
267	1.000000	0.001791	-0.001381	0.003172	0.003514
268	1.000000	0.004464	-0.000704	0.005168	0.001306
269	1.000000	0.000208	0.000044	0.000164	0.001730
270	1.000000	-0.000304	0.000047	-0.000351	-0.000371
271	1.000000	-0.000019	-0.000014	-0.000005	-0.000003
272	1.000000	0.000608	-0.000039	0.000647	0.003137
273	1.000000	-0.000454	-0.000048	-0.000406	0.002620
274	1.000000	-0.000798	-0.000054	-0.000744	-0.001009
275	1.000000	0.000034	0.000076	-0.000042	-0.000088
276	1.000000	0.000180	-0.000059	0.000239	0.000796
277	1.000000	-0.000095	-0.000281	0.000186	-0.000587
278	1.000000	0.000897	0.000471	0.000426	-0.005971
279	1.000000	-0.001714	-0.001650	-0.000064	-0.001446
280	1.000000	-0.000400	-0.001708	0.001308	-0.002965
281	1.000000	-0.001613	-0.000090	-0.001523	-0.000419
282	1.000000	-0.000090	0.000335	-0.000425	-0.000638
283	1.000000	0.001103	0.000279	0.000824	0.003146
284	1.000000	0.000309	0.000061	0.000249	0.000292
285	1.000000	0.000007	-0.000121	0.000128	0.000930
286	1.000000	0.000016	-0.001267	0.001283	-0.001759
287	1.000000	0.000448	0.000104	0.000344	0.001177
288	1.000000	0.000436	0.000078	0.000358	0.000945
289	1.000000	0.000005	-0.000553	0.000558	0.001468
290	1.000000	0.000976	0.000809	0.000166	0.002026
291	1.000000	0.001384	-0.000313	0.001696	0.001485
292	1.000000	0.000074	0.000008	0.000066	0.000600
293	1.000000	0.000567	0.000055	0.000512	0.001838
294	1.000000	-0.000089	0.000235	-0.000324	0.003377
295	1.000000	0.000299	0.000161	0.000138	0.001259
296	1.000000	-0.000088	0.000273	-0.000360	0.000130
297	1.000000	-0.000058	0.000194	-0.000251	0.000935

298	1.000000	0.001651	-0.000096	0.001746	0.003001
299	1.000000	0.001228	0.000032	0.001196	0.001226
300	1.000000	0.000232	-0.000344	0.000576	0.002795
301	1.000000	-0.000004	0.000272	-0.000275	-0.000222
302	1.000000	-0.000004	0.000017	-0.000021	0.000739
303	1.000000	0.000274	-0.000144	0.000419	-0.003254
304	1.000000	-0.000651	-0.000090	-0.000561	-0.000782
305	1.000000	0.001041	-0.000275	0.001316	-0.000396
306	1.000000	0.000322	-0.000060	0.000382	-0.000269
307	1.000000	0.000066	0.000008	0.000058	0.000047
308	1.000000	-0.001006	-0.000184	-0.000822	-0.002070
309	1.000000	0.000042	0.000070	-0.000027	-0.002806
310	1.000000	-0.000040	-0.000045	0.000005	-0.000664
311	1.000000	-0.000018	0.000010	-0.000028	0.001527
312	1.000000	0.000069	0.000092	-0.000023	0.000156
313	1.000000	0.000053	0.000004	0.000049	0.000278
314	1.000000	0.000098	0.000012	0.000086	0.000399
315	1.000000	-0.000010	0.000033	-0.000044	-0.000422
316	1.000000	0.000173	0.000391	-0.000217	0.001886
317	1.000000	-0.001690	-0.003818	0.002129	-0.011032
318	1.000000	0.000053	0.000620	-0.000566	-0.000028
319	1.000000	-0.000249	-0.000473	0.000224	-0.000922
320	1.000000	-0.000450	0.000251	-0.000701	0.001602
321	1.000000	-0.001503	-0.001581	0.000078	0.000597
322	1.000000	-0.000188	-0.003616	0.003428	0.003530
323	1.000000	0.003780	0.002240	0.001540	0.001997
324	1.000000	0.001250	0.000757	0.000493	0.005813
325	1.000000	0.000369	0.000184	0.000184	0.000252
326	1.000000	0.000341	-0.000104	0.000445	0.000161
327	1.000000	-0.000031	-0.000021	-0.000010	-0.000659
328	1.000000	0.000708	0.000289	0.000419	-0.001025
329	1.000000	0.000045	0.000133	-0.000088	0.001572
330	1.000000	0.000168	-0.000027	0.000195	0.000222
331	1.000000	-0.000094	-0.000055	-0.000039	0.000445
332	1.000000	-0.000123	0.000271	-0.000394	-0.000028
333	1.000000	-0.000977	-0.001886	0.000910	0.002024
334	1.000000	0.000120	0.000314	-0.000194	0.001773
335	1.000000	0.000434	0.000276	0.000158	0.001843
336	1.000000	-0.000488	-0.000458	-0.000029	0.006199
337	1.000000	-0.000356	0.000536	-0.000892	-0.000771
338	1.000000	0.000001	0.000084	-0.000083	0.000461
339	1.000000	0.000168	0.000398	-0.000230	-0.001079
340	1.000000	-0.002060	-0.000788	-0.001271	-0.001958
341	1.000000	-0.001112	0.000084	-0.001197	-0.001950

342	1.000000	0.000102	-0.000038	0.000140	0.002374
343	1.000000	0.000215	-0.000041	0.000256	-0.000463
344	1.000000	0.002701	0.001137	0.001565	-0.006747
345	1.000000	-0.000000	-0.000017	0.000017	-0.003988
346	1.000000	0.000186	0.000577	-0.000391	-0.001241
347	1.000000	-0.001216	0.001509	-0.002725	-0.003076
348	1.000000	0.000031	0.000187	-0.000157	-0.001354
349	1.000000	-0.000022	0.000312	-0.000334	-0.000918
350	1.000000	-0.000766	0.000761	-0.001527	-0.005954
351	1.000000	-0.000010	0.000001	-0.000011	-0.000132
352	1.000000	-0.000243	0.000375	-0.000619	-0.000653
353	1.000000	-0.001244	-0.000139	-0.001105	-0.004078
354	1.000000	-0.000046	0.000002	-0.000048	-0.000072
355	1.000000	-0.000083	-0.000043	-0.000040	-0.000216
356	1.000000	-0.000132	0.001369	-0.001501	0.000359
357	1.000000	-0.000065	0.001946	-0.002012	0.000771
358	1.000000	-0.001671	0.000483	-0.002154	-0.001460
359	1.000000	-0.000113	0.000063	-0.000176	-0.000178
360	1.000000	-0.000393	0.004582	-0.004974	-0.002777
361	1.000000	-0.000349	0.002374	-0.002722	0.001940
362	1.000000	-0.000012	0.000068	-0.000079	0.000393
363	1.000000	0.001734	0.001085	0.000650	-0.015657
364	1.000000	0.000021	0.000944	-0.000924	0.003629
365	1.000000	0.000558	-0.000520	0.001079	-0.005614
366	1.000000	-0.000239	0.002286	-0.002526	-0.004750
367	1.000000	0.000264	0.000993	-0.000729	-0.000984
368	1.000000	0.001051	-0.002363	0.003415	0.004148
369	1.000000	-0.000596	-0.001187	0.000591	-0.000523
370	1.000000	-0.000652	-0.000259	-0.000393	0.001792
371	1.000000	0.001093	-0.000254	0.001347	-0.007062
372	1.000000	0.000633	0.000119	0.000514	-0.005219
373	1.000000	0.000949	0.000232	0.000717	-0.002954
374	1.000000	0.000173	0.000079	0.000095	-0.004871
375	1.000000	0.001486	0.000523	0.000963	-0.003542
376	1.000000	0.001353	-0.000029	0.001382	-0.007280
377	1.000000	0.001436	-0.000246	0.001682	-0.006652
378	1.000000	0.004373	0.000763	0.003610	-0.016662
379	1.000000	0.004743	0.001055	0.003688	-0.027907
380	1.000000	0.004949	0.004881	0.000067	-0.051422
381	1.000000	0.001280	0.003183	-0.001903	-0.021537
382	1.000000	-0.002688	-0.000953	-0.001736	-0.013671
383	1.000000	0.005587	-0.000296	0.005883	-0.005478
384	1.000000	0.000026	-0.000036	0.000062	-0.000707
385	1.000000	0.004825	0.000954	0.003871	-0.011647

386	1.000000	0.001188	0.000374	0.000813	-0.008553
387	1.000000	-0.000031	0.000018	-0.000050	-0.004301
388	1.000000	-0.002388	0.004447	-0.006835	-0.048420
389	1.000000	-0.003806	0.000155	-0.003961	-0.002654
390	0.000000	0.000000	0.000000	0.000000	0.000000
391	0.000000	0.000000	0.000000	0.000000	0.000000
392	0.000000	0.000000	0.000000	0.000000	0.000000
393	0.000000	0.000000	0.000000	0.000000	0.000000

......

Sum:	389.000000	0.132294	-0.006926	0.139221	-0.186676

**** Result for beta electrons ****

Orb.	Occ.	d	b	d - b	r
1	1.000000	-0.000000	-0.000000	-0.000000	-0.000000
2	1.000000	-0.000015	0.000028	-0.000043	0.000011
3	1.000000	-0.000000	-0.000000	-0.000000	-0.000000
4	1.000000	-0.000000	-0.000000	-0.000000	-0.000000
5	1.000000	-0.000001	0.000000	-0.000001	0.000000
6	1.000000	-0.000000	0.000002	-0.000002	-0.000000
7	1.000000	-0.000000	-0.000000	-0.000000	-0.000000
8	1.000000	-0.000599	-0.001563	0.000963	-0.000877
9	1.000000	-0.000000	-0.000000	-0.000000	-0.000000
10	1.000000	-0.000000	-0.000000	-0.000000	-0.000000
11	1.000000	-0.000000	-0.000000	-0.000000	-0.000000
12	1.000000	-0.000206	-0.000132	-0.000075	-0.000139
13	1.000000	-0.000064	-0.000098	0.000034	-0.000039
14	1.000000	-0.000018	-0.000033	0.000015	-0.000006
15	1.000000	-0.000002	-0.000001	-0.000001	-0.000000
16	1.000000	-0.000000	-0.000000	-0.000000	-0.000000
17	1.000000	-0.000001	-0.000000	-0.000001	-0.000000
18	1.000000	-0.000002	-0.000001	-0.000001	-0.000000
19	1.000000	-0.000008	-0.000002	-0.000006	-0.000001
20	1.000000	-0.000021	-0.000047	0.000026	-0.000003
21	1.000000	-0.000024	0.000002	-0.000027	-0.000002
22	1.000000	-0.000021	-0.000073	0.000052	0.000003
23	1.000000	-0.000000	-0.000000	-0.000000	-0.000000
24	1.000000	-0.000000	-0.000000	-0.000000	-0.000000
25	1.000000	-0.000074	-0.000183	0.000110	-0.000023
26	1.000000	-0.000001	-0.000000	-0.000001	-0.000000
27	1.000000	-0.000001	-0.000000	-0.000000	-0.000000
28	1.000000	-0.000038	-0.000167	0.000129	-0.000014
29	1.000000	-0.000055	-0.000028	-0.000027	-0.000005

30	1.000000	-0.000000	-0.000000	-0.000000	-0.000000
31	1.000000	-0.000000	-0.000000	-0.000000	-0.000000
32	1.000000	-0.000000	-0.000000	-0.000000	-0.000000
33	1.000000	-0.000000	-0.000000	-0.000000	-0.000000
34	1.000000	-0.000005	-0.000001	-0.000004	-0.000001
35	1.000000	-0.000000	-0.000000	-0.000000	-0.000000
36	1.000000	-0.000001	-0.000001	0.000000	-0.000000
37	1.000000	-0.000019	0.000001	-0.000021	0.000000
38	1.000000	-0.000045	0.000009	-0.000055	-0.000003
39	1.000000	-0.000000	-0.000000	-0.000000	-0.000000
40	1.000000	-0.000000	-0.000000	-0.000000	-0.000000
41	1.000000	-0.000000	-0.000000	-0.000000	-0.000000
42	1.000000	-0.000000	-0.000000	-0.000000	-0.000000
43	1.000000	-0.000000	-0.000000	-0.000000	-0.000000
44	1.000000	-0.000018	-0.000007	-0.000011	-0.000004
45	1.000000	-0.000006	-0.000001	-0.000005	-0.000001
46	1.000000	-0.000001	-0.000000	-0.000001	-0.000000
47	1.000000	-0.000001	-0.000001	-0.000000	-0.000000
48	1.000000	-0.000000	-0.000000	-0.000000	-0.000000
49	1.000000	0.000744	-0.000030	0.000775	-0.000003
50	1.000000	-0.000003	-0.000001	-0.000002	-0.000001
51	1.000000	0.000003	-0.000001	0.000003	-0.000000
52	1.000000	-0.001675	-0.000090	-0.001585	-0.000205
53	1.000000	-0.000003	-0.000002	-0.000002	-0.000001
54	1.000000	-0.000000	-0.000000	-0.000000	-0.000000
55	1.000000	-0.000001	-0.000000	-0.000000	-0.000000
56	1.000000	-0.000204	-0.000013	-0.000192	-0.000021
57	1.000000	-0.000001	-0.000001	-0.000000	-0.000000
58	1.000000	-0.000532	-0.000033	-0.000498	-0.000031
59	1.000000	-0.000004	-0.000002	-0.000002	-0.000002
60	1.000000	-0.000324	-0.000013	-0.000311	-0.000018
61	1.000000	-0.000010	-0.000002	-0.000008	-0.000002
62	1.000000	-0.000003	-0.000001	-0.000001	-0.000001
63	1.000000	-0.000001	-0.000001	-0.000000	-0.000000
64	1.000000	-0.000001	-0.000000	-0.000000	-0.000000
65	1.000000	-0.000000	-0.000000	-0.000000	-0.000000
66	1.000000	-0.000000	-0.000000	-0.000000	-0.000000
67	1.000000	-0.000184	-0.000019	-0.000165	-0.000012
68	1.000000	-0.000000	-0.000000	0.000000	-0.000000
69	1.000000	-0.000099	-0.000003	-0.000096	-0.000005
70	1.000000	-0.000003	-0.000000	-0.000003	-0.000000
71	1.000000	-0.000001	-0.000001	-0.000001	-0.000000
72	1.000000	-0.000001	-0.000000	-0.000000	-0.000000
73	1.000000	-0.000139	-0.000010	-0.000129	-0.000010

74	1.000000	-0.000000	-0.000000	-0.000000	-0.000000
75	1.000000	-0.000002	-0.000001	-0.000001	-0.000000
76	1.000000	-0.000002	-0.000002	-0.000001	-0.000001
77	1.000000	-0.000269	-0.000028	-0.000240	-0.000026
78	1.000000	-0.000001	-0.000001	-0.000001	-0.000000
79	1.000000	-0.000001	-0.000000	-0.000000	-0.000000
80	1.000000	-0.000920	-0.000052	-0.000868	-0.000071
81	1.000000	-0.000002	-0.000001	-0.000001	-0.000000
82	1.000000	-0.000003	-0.000001	-0.000001	-0.000001
83	1.000000	-0.000001	-0.000000	-0.000001	-0.000000
84	1.000000	-0.000001	-0.000001	-0.000000	-0.000000
85	1.000000	-0.000387	-0.000027	-0.000360	-0.000042
86	1.000000	-0.000004	-0.000001	-0.000002	-0.000001
87	1.000000	-0.000166	-0.000005	-0.000161	-0.000007
88	1.000000	-0.000018	-0.000005	-0.000012	-0.000003
89	1.000000	0.000000	-0.000000	0.000001	-0.000000
90	1.000000	-0.000000	-0.000000	0.000000	-0.000000
91	1.000000	-0.000001	-0.000000	-0.000000	-0.000000
92	1.000000	-0.000000	-0.000000	-0.000000	-0.000000
93	1.000000	-0.000001	-0.000001	-0.000000	-0.000000
94	1.000000	-0.000003	-0.000002	-0.000001	-0.000001
95	1.000000	-0.000001	-0.000000	-0.000001	-0.000000
96	1.000000	-0.000001	-0.000000	-0.000001	-0.000000
97	1.000000	-0.000001	-0.000001	-0.000001	-0.000000
98	1.000000	-0.000096	-0.000006	-0.000090	-0.000008
99	1.000000	-0.000002	-0.000001	-0.000002	-0.000000
100	1.000000	-0.000000	-0.000001	0.000000	-0.000000
101	1.000000	-0.000000	-0.000000	0.000000	-0.000000
102	1.000000	-0.000003	-0.000002	-0.000001	-0.000001
103	1.000000	-0.000000	-0.000000	0.000000	-0.000000
104	1.000000	-0.000151	-0.000045	-0.000106	-0.000028
105	1.000000	-0.000013	0.000034	-0.000047	0.000002
106	1.000000	-0.000001	-0.000000	-0.000001	-0.000000
107	1.000000	-0.000005	-0.000002	-0.000003	-0.000001
108	1.000000	-0.000065	-0.000009	-0.000056	-0.000009
109	1.000000	-0.000012	-0.000002	-0.000010	-0.000002
110	1.000000	-0.000029	-0.000004	-0.000025	-0.000004
111	1.000000	-0.000002	-0.000001	-0.000001	-0.000000
112	1.000000	-0.000024	-0.000006	-0.000018	-0.000004
113	1.000000	-0.000000	-0.000000	-0.000000	-0.000000
114	1.000000	-0.000022	0.000021	-0.000042	0.000001
115	1.000000	-0.000000	-0.000000	-0.000000	-0.000000
116	1.000000	-0.000000	-0.000000	-0.000000	-0.000000
117	1.000000	-0.000007	0.000007	-0.000014	0.000001

118	1.000000	-0.000008	0.000065	-0.000074	-0.000006
119	1.000000	-0.000000	-0.000000	-0.000000	-0.000000
120	1.000000	-0.000000	-0.000000	-0.000000	-0.000000
121	1.000000	-0.000000	-0.000000	-0.000000	-0.000000
122	1.000000	-0.000001	-0.000000	-0.000000	-0.000000
123	1.000000	-0.000000	-0.000000	-0.000000	-0.000000
124	1.000000	-0.000000	-0.000000	-0.000000	-0.000000
125	1.000000	-0.000009	-0.000008	-0.000000	-0.000001
126	1.000000	-0.000073	-0.000024	-0.000048	-0.000011
127	1.000000	-0.000027	-0.000017	-0.000011	-0.000005
128	1.000000	-0.000013	-0.000003	-0.000010	-0.000002
129	1.000000	-0.000044	-0.000099	0.000055	-0.000019
130	1.000000	-0.000142	-0.000069	-0.000073	-0.000025
131	1.000000	-0.000000	-0.000000	-0.000000	-0.000000
132	1.000000	-0.000225	-0.003728	0.003503	-0.002249
133	1.000000	-0.000000	-0.000000	-0.000000	-0.000000
134	1.000000	-0.000000	-0.000000	-0.000000	-0.000000
135	1.000000	-0.000000	-0.000000	-0.000000	-0.000000
136	1.000000	-0.000007	-0.000147	0.000140	-0.000078
137	1.000000	-0.000002	-0.000033	0.000030	-0.000026
138	1.000000	-0.000002	-0.000070	0.000068	-0.000020
139	1.000000	-0.000005	-0.000000	-0.000005	-0.000000
140	1.000000	-0.000001	-0.000000	-0.000001	-0.000000
141	1.000000	-0.000003	0.000000	-0.000003	-0.000000
142	1.000000	-0.000094	-0.000430	0.000336	-0.000187
143	1.000000	-0.000067	0.000215	-0.000283	0.000084
144	1.000000	-0.000001	-0.000000	-0.000000	-0.000000
145	1.000000	-0.000002	0.000001	-0.000003	0.000000
146	1.000000	-0.000001	-0.000000	-0.000001	-0.000000
147	1.000000	-0.000001	-0.000001	-0.000001	-0.000000
148	1.000000	-0.000134	0.000250	-0.000384	-0.000015
149	1.000000	-0.000473	0.001647	-0.002119	0.000300
150	1.000000	-0.000019	-0.000745	0.000726	0.000178
151	1.000000	0.001269	0.000018	0.001251	0.000368
152	1.000000	0.000009	-0.000747	0.000756	-0.000134
153	1.000000	0.003996	-0.000286	0.004282	0.001137
154	1.000000	0.001371	-0.000055	0.001426	0.000354
155	1.000000	0.003223	-0.000159	0.003382	0.000823
156	1.000000	-0.000707	-0.002649	0.001943	-0.000869
157	1.000000	0.000771	-0.000020	0.000791	0.000185
158	1.000000	-0.000005	-0.000086	0.000082	-0.000006
159	1.000000	0.003726	-0.000089	0.003815	0.000879
160	1.000000	0.000582	-0.000012	0.000594	0.000138
161	1.000000	0.007341	-0.000208	0.007549	0.001748

162	1.000000	0.002482	-0.000026	0.002507	0.000412
163	1.000000	-0.000169	-0.000697	0.000528	-0.000131
164	1.000000	0.001069	-0.000010	0.001079	0.000381
165	1.000000	0.001407	-0.000037	0.001445	0.000385
166	1.000000	0.003805	-0.000031	0.003836	0.000704
167	1.000000	-0.000000	-0.000000	-0.000000	0.000000
168	1.000000	-0.000000	-0.000000	-0.000000	-0.000000
169	1.000000	0.002355	-0.000086	0.002441	0.000716
170	1.000000	0.001698	-0.000044	0.001742	0.000456
171	1.000000	0.001192	-0.000042	0.001234	0.000308
172	1.000000	0.007533	-0.000856	0.008389	0.002620
173	1.000000	0.000280	-0.000002	0.000282	-0.000480
174	1.000000	-0.000090	-0.000283	0.000193	-0.000115
175	1.000000	-0.000001	-0.000001	0.000000	-0.000000
176	1.000000	-0.000001	-0.000002	0.000001	0.000000
177	1.000000	-0.000008	-0.000050	0.000042	0.000012
178	1.000000	0.000448	-0.000249	0.000697	0.001997
179	1.000000	-0.000240	-0.000222	-0.000018	0.000438
180	1.000000	0.001599	-0.000121	0.001720	-0.000313
181	1.000000	0.000464	-0.000031	0.000494	-0.000151
182	1.000000	0.004772	-0.000162	0.004935	0.001215
183	1.000000	0.001001	-0.000016	0.001017	0.000818
184	1.000000	0.000798	-0.000044	0.000843	0.000978
185	1.000000	-0.000348	-0.000171	-0.000176	-0.000883
186	1.000000	-0.000197	-0.000407	0.000210	-0.000671
187	1.000000	0.009135	-0.000284	0.009418	0.002773
188	1.000000	0.001285	-0.000043	0.001328	0.000307
189	1.000000	0.000281	-0.000002	0.000284	0.000220
190	1.000000	0.001693	-0.000023	0.001716	0.000377
191	1.000000	-0.000012	-0.000178	0.000167	0.000002
192	1.000000	0.000015	-0.000332	0.000347	0.000130
193	1.000000	0.004147	-0.000087	0.004233	0.001444
194	1.000000	0.001378	-0.000019	0.001396	0.000586
195	1.000000	0.003531	-0.000143	0.003674	0.001278
196	1.000000	0.000747	-0.000012	0.000758	0.000434
197	1.000000	-0.000025	-0.000001	-0.000024	0.000048
198	1.000000	0.000800	-0.000011	0.000810	0.000845
199	1.000000	0.000437	-0.000049	0.000487	0.000496
200	1.000000	0.002153	-0.000283	0.002436	0.000713
201	1.000000	-0.000001	0.000001	-0.000001	0.000000
202	1.000000	-0.000019	-0.000330	0.000312	0.000014
203	1.000000	-0.000001	-0.000013	0.000012	-0.000000
204	1.000000	-0.000000	-0.000000	-0.000000	-0.000000
205	1.000000	-0.000074	-0.000485	0.000411	0.000139

206	1.000000	-0.000000	0.000000	-0.000000	-0.000000
207	1.000000	-0.000295	-0.000362	0.000067	0.001105
208	1.000000	0.000152	-0.000139	0.000291	0.000023
209	1.000000	0.003866	-0.000133	0.004000	0.001229
210	1.000000	0.001541	-0.000008	0.001549	0.000717
211	1.000000	-0.000039	-0.000152	0.000113	0.000011
212	1.000000	0.005261	-0.000352	0.005613	0.002806
213	1.000000	0.000029	0.000011	0.000018	0.000393
214	1.000000	0.001306	-0.000193	0.001500	0.000865
215	1.000000	0.000061	0.000070	-0.000009	0.000134
216	1.000000	0.000211	-0.000014	0.000225	0.000162
217	1.000000	0.000120	-0.000006	0.000126	0.000146
218	1.000000	0.000968	-0.000021	0.000990	0.001288
219	1.000000	0.000643	-0.000014	0.000656	0.000866
220	1.000000	0.000028	0.000001	0.000027	0.000342
221	1.000000	-0.000064	-0.000075	0.000011	-0.000005
222	1.000000	0.001412	-0.000533	0.001945	0.000831
223	1.000000	-0.000457	-0.000879	0.000422	-0.000113
224	1.000000	-0.000386	-0.001079	0.000692	0.000209
225	1.000000	-0.000022	-0.000200	0.000178	0.001034
226	1.000000	-0.000015	-0.000031	0.000017	0.000003
227	1.000000	-0.000101	-0.000362	0.000260	0.000281
228	1.000000	0.000137	-0.000011	0.000148	0.000270
229	1.000000	0.001131	-0.000042	0.001174	0.001487
230	1.000000	0.001178	-0.000099	0.001277	0.002551
231	1.000000	0.000278	-0.000012	0.000290	0.000506
232	1.000000	-0.000208	-0.000653	0.000445	0.000301
233	1.000000	-0.000064	0.000020	-0.000084	0.000256
234	1.000000	-0.000044	-0.000265	0.000221	0.000718
235	1.000000	-0.000697	-0.000031	-0.000667	0.000867
236	1.000000	0.001305	0.000004	0.001301	0.002490
237	1.000000	-0.000000	0.000001	-0.000002	0.000002
238	1.000000	0.002428	-0.000220	0.002647	0.002181
239	1.000000	0.000414	-0.000038	0.000451	0.000385
240	1.000000	0.000095	0.000004	0.000091	0.000201
241	1.000000	0.000088	0.000012	0.000076	0.000605
242	1.000000	-0.000030	0.000249	-0.000279	0.001095
243	1.000000	-0.000001	-0.000002	0.000000	0.000005
244	1.000000	0.000084	-0.000027	0.000111	0.000066
245	1.000000	0.001348	-0.000304	0.001652	0.001776
246	1.000000	0.000811	-0.000084	0.000894	0.001528
247	1.000000	-0.000173	-0.000500	0.000327	-0.000178
248	1.000000	-0.000024	-0.000042	0.000019	0.000066
249	1.000000	-0.000123	-0.000234	0.000111	0.000733

250	1.000000	0.000257	-0.000783	0.001039	0.003318
251	1.000000	0.000006	-0.000065	0.000071	0.000247
252	1.000000	0.001343	-0.001205	0.002547	0.003982
253	1.000000	0.004182	-0.000241	0.004423	0.004616
254	1.000000	0.000179	-0.000009	0.000188	0.000569
255	1.000000	0.001010	-0.000187	0.001197	0.001926
256	1.000000	0.000753	-0.000016	0.000769	0.001992
257	1.000000	0.000008	-0.000007	0.000015	-0.000012
258	1.000000	-0.000092	0.000113	-0.000205	0.000209
259	1.000000	-0.000382	-0.000584	0.000202	0.001448
260	1.000000	-0.000019	-0.000111	0.000092	0.000754
261	1.000000	-0.000001	0.000024	-0.000025	0.001808
262	1.000000	0.000078	-0.000505	0.000583	0.007019
263	1.000000	0.000027	-0.000099	0.000127	0.000502
264	1.000000	-0.000065	0.000042	-0.000107	0.000168
265	1.000000	-0.000089	0.000026	-0.000115	0.000288
266	1.000000	0.000146	-0.000691	0.000838	0.001526
267	1.000000	0.003408	-0.001170	0.004578	0.003353
268	1.000000	0.003381	-0.000381	0.003762	0.000921
269	1.000000	0.000281	0.000070	0.000211	0.001372
270	1.000000	-0.000133	0.000055	-0.000188	0.000256
271	1.000000	0.000660	-0.000020	0.000680	0.002942
272	1.000000	-0.000011	-0.000007	-0.000004	-0.000003
273	1.000000	-0.000243	0.000048	-0.000291	0.000650
274	1.000000	-0.001067	-0.000083	-0.000985	0.001040
275	1.000000	-0.000125	0.000164	-0.000290	0.000235
276	1.000000	0.000154	-0.000064	0.000218	0.000753
277	1.000000	0.000772	-0.000105	0.000877	-0.006164
278	1.000000	-0.000551	-0.000878	0.000327	-0.002184
279	1.000000	-0.000965	-0.000443	-0.000522	-0.000351
280	1.000000	-0.000944	-0.002263	0.001319	-0.004021
281	1.000000	-0.000868	0.000353	-0.001221	-0.000026
282	1.000000	-0.000076	0.000312	-0.000388	-0.000868
283	1.000000	0.001062	0.000337	0.000724	0.002789
284	1.000000	0.000303	0.000048	0.000255	0.000208
285	1.000000	0.000034	0.000046	-0.000012	0.001066
286	1.000000	-0.000020	-0.001426	0.001406	-0.001470
287	1.000000	0.000426	0.000117	0.000309	0.001015
288	1.000000	0.000413	0.000057	0.000357	0.000887
289	1.000000	0.000039	-0.000540	0.000579	0.001131
290	1.000000	0.002357	0.000515	0.001842	0.002461
291	1.000000	0.000175	0.000033	0.000142	0.000625
292	1.000000	0.000528	0.000083	0.000446	0.001635
293	1.000000	0.000011	-0.000294	0.000305	0.003449

294	1.000000	0.000157	0.000028	0.000129	0.002502
295	1.000000	-0.000099	0.000670	-0.000769	0.000746
296	1.000000	-0.000194	0.000574	-0.000767	0.000359
297	1.000000	0.002467	-0.000162	0.002629	0.001951
298	1.000000	0.000659	-0.000038	0.000697	0.001860
299	1.000000	0.000554	-0.000449	0.001003	0.003738
300	1.000000	-0.000068	0.000055	-0.000123	0.000372
301	1.000000	0.000520	0.000185	0.000335	0.000287
302	1.000000	-0.000249	-0.000180	-0.000069	-0.002119
303	1.000000	-0.000785	-0.000165	-0.000620	-0.001660
304	1.000000	0.000599	0.000008	0.000591	-0.001450
305	1.000000	0.000727	-0.000021	0.000748	-0.001102
306	1.000000	-0.000454	-0.000122	-0.000332	-0.001192
307	1.000000	-0.000404	0.000042	-0.000447	-0.003369
308	1.000000	-0.000001	0.000020	-0.000022	-0.000120
309	1.000000	0.000131	0.000143	-0.000012	0.001725
310	1.000000	-0.000191	-0.000102	-0.000088	-0.001046
311	1.000000	0.000056	0.000003	0.000053	0.000219
312	1.000000	0.000089	0.000001	0.000089	0.000394
313	1.000000	-0.000012	0.000014	-0.000026	-0.000113
314	1.000000	0.000032	0.000283	-0.000251	0.001887
315	1.000000	0.000003	-0.000002	0.000005	-0.000034
316	1.000000	0.000083	0.000459	-0.000376	-0.001788
317	1.000000	-0.001336	-0.002879	0.001543	-0.006194
318	1.000000	-0.001392	-0.002702	0.001310	-0.003231
319	1.000000	-0.000065	0.000036	-0.000102	0.000115
320	1.000000	-0.000577	0.000608	-0.001185	0.004079
321	1.000000	-0.000533	-0.000576	0.000043	0.000575
322	1.000000	0.002067	-0.002800	0.004867	0.006198
323	1.000000	0.001007	0.001023	-0.000016	0.000636
324	1.000000	0.000671	0.001067	-0.000396	0.004015
325	1.000000	0.000972	0.000391	0.000581	-0.000841
326	1.000000	0.001170	0.000319	0.000851	-0.000149
327	1.000000	0.000302	0.000107	0.000195	0.000719
328	1.000000	-0.000307	-0.000102	-0.000205	0.001234
329	1.000000	0.000737	0.000158	0.000579	-0.000757
330	1.000000	-0.000101	0.000012	-0.000113	0.000222
331	1.000000	-0.000003	-0.000129	0.000126	-0.001105
332	1.000000	-0.001103	-0.000929	-0.000174	0.001373
333	1.000000	0.000177	-0.000261	0.000438	0.003656
334	1.000000	-0.000313	-0.000150	-0.000163	0.000092
335	1.000000	-0.000194	-0.000639	0.000445	0.005160
336	1.000000	-0.000194	0.000766	-0.000959	-0.001051
337	1.000000	0.000010	-0.000003	0.000012	0.000087

338	1.000000	0.000241	0.000409	-0.000168	-0.000066
339	1.000000	-0.001517	-0.000545	-0.000972	-0.001852
340	1.000000	-0.001874	0.000032	-0.001906	-0.003630
341	1.000000	0.000020	-0.000086	0.000106	0.003234
342	1.000000	0.000705	0.000235	0.000470	-0.000725
343	1.000000	0.000029	0.000085	-0.000056	-0.000620
344	1.000000	0.003717	0.001268	0.002449	-0.007376
345	1.000000	-0.000582	-0.000049	-0.000534	-0.002311
346	1.000000	0.000152	0.001295	-0.001143	-0.001771
347	1.000000	-0.000955	0.001028	-0.001983	-0.003571
348	1.000000	-0.000090	0.000178	-0.000268	-0.000911
349	1.000000	-0.000519	0.000897	-0.001416	-0.004484
350	1.000000	-0.000179	0.000406	-0.000585	-0.002573
351	1.000000	-0.000799	0.000310	-0.001109	-0.002943
352	1.000000	-0.001194	0.000060	-0.001255	-0.002671
353	1.000000	-0.000053	0.000011	-0.000064	-0.000093
354	1.000000	-0.000142	0.000055	-0.000197	-0.000313
355	1.000000	-0.000154	0.001883	-0.002037	-0.000745
356	1.000000	-0.000075	0.002332	-0.002408	-0.000538
357	1.000000	-0.000143	0.000123	-0.000266	0.000016
358	1.000000	-0.000961	0.000223	-0.001184	0.000020
359	1.000000	-0.001285	0.004156	-0.005441	0.001689
360	1.000000	0.000082	0.003245	-0.003163	-0.002081
361	1.000000	-0.000072	-0.000231	0.000159	-0.000430
362	1.000000	0.001492	0.001141	0.000351	-0.015864
363	1.000000	-0.000406	0.001253	-0.001660	0.000533
364	1.000000	-0.000365	0.000631	-0.000996	-0.004772
365	1.000000	0.000987	0.001979	-0.000992	-0.006402
366	1.000000	0.000129	0.001245	-0.001116	-0.001137
367	1.000000	0.001052	-0.002503	0.003555	0.003743
368	1.000000	-0.001012	-0.001194	0.000182	0.000108
369	1.000000	0.000423	-0.000048	0.000471	0.002437
370	1.000000	0.000387	-0.000294	0.000681	-0.004141
371	1.000000	0.000232	0.000483	-0.000252	-0.001362
372	1.000000	0.000369	0.000083	0.000286	-0.005912
373	1.000000	0.000520	-0.000143	0.000663	-0.006952
374	1.000000	0.001397	-0.000173	0.001571	-0.008511
375	1.000000	0.007522	0.000871	0.006651	-0.022533
376	1.000000	0.000380	0.000426	-0.000046	-0.008518
377	1.000000	-0.000147	-0.000121	-0.000027	-0.001170
378	1.000000	0.006135	0.001126	0.005008	-0.024640
379	1.000000	0.004692	0.005995	-0.001303	-0.053404
380	1.000000	-0.000350	0.003654	-0.004003	-0.019807
381	1.000000	-0.003182	-0.000686	-0.002497	-0.015278

382	1.000000	0.008259	0.000696	0.007563	-0.012098
383	1.000000	0.001786	0.000137	0.001650	-0.003345
384	1.000000	0.000249	-0.000029	0.000279	-0.008601
385	1.000000	0.000064	0.000015	0.000050	-0.001300
386	1.000000	0.000526	0.000217	0.000309	-0.002821
387	1.000000	-0.003283	0.004990	-0.008272	-0.051031
388	0.000000	0.000000	0.000000	0.000000	0.000000
389	0.000000	0.000000	0.000000	0.000000	0.000000
390	0.000000	0.000000	0.000000	0.000000	0.000000
391	0.000000	0.000000	0.000000	0.000000	0.000000
......					
Sum:	387.000000	0.134947	-0.000894	0.135841	-0.198932

Result for all electrons:

d= 0.267241   b= -0.007820   d - b = 0.275061   r= -0.385608

Supplementary Table 3. Elemental analysis for $Cu_2(L)(py)_4$ and $\{[Cu_4(Li^+@C_{60})L(py)_4](NTf_2)(hexane)\}_n$.

Compounds	C (%) Cal. / Exp.	H (%) Cal. / Exp.	N (%) Cal. / Exp.
$Cu_2(L)(py)_4$	40.49 / 40.46	3.85 / 3.86	12.59 / 12.47
$\{[Cu_4(Li^+@C_{60})L(py)_4](NTf_2)(hexane)\}_n$	55.76 / 55.34	2.29 / 1.96	5.97 / 5.71
